# 3-months elimination diet in childhood EoE: nutritional and immunological aspects

**DOI:** 10.1186/2045-7022-3-S3-P4

**Published:** 2013-07-25

**Authors:** D Colson, N Kalach, P Soulaines, B Michaud, L Chatenoud, C Dupont

**Affiliations:** 1 Nutritia Nutrition Clinique, Saint Ouen, France; 2 Institut Catholique de Lille, Lille, France; 3 Hopital Necker Enfants-Malades, Paris, France; 4 Laboratoire d'Immunologie Biologie Inserm U 1013, Hopital Necker Enfants-Malades, Paris, France; 5 U1013, Hopital Necker Enfants-Malades, Paris, France

## Background

The use of dietary treatment in eosinophilic esophagitis (EoE) in children is regularly described in literature. The aim of the study was to evaluate anthropomorphic and biological parameters in EoE children before and after elimination diet.

## Methods

A cohort of 87 consecutive patients with EoE (>15 eosinophils/hpf in esophageal biopsies) was retrospectively analyzed. All children followed the so-called modified six-food elimination diet (SFED), excluding 6 main offending foods retrieved together with those eliciting positive SPT and APT, supplemented with a nutritional support by an amino acid formula. Patients had the following treatment sequence: endoscopy/clinical/biological assessment, 3 months elimination diet and second endoscopy/clinical/ biological assessment.

## Results

Analysis included 49 patients. At enrolment, BMI was significantly lower in females than in males (p<0.05), and total circulating IgG and IgM levels were higher in females (p<0.05). Patients having recovered at the end of the study happened to exhibit at enrolment a BMI significantly higher than those having shown only partial or no recovery (p<0.05). Elimination diet led to complete recovery (no symptoms & <5 eosinophils/hpf) in 53% cases. Following the elimination diet, BMI remained lower in females than in males. Also, blood eosinophils counts (p<0.0001), IgG (p0.003) and IgM levels (p<0.05) showed a significant decrease as well as specific IgE titers against CMP (p<0.02) and egg (p<0.02), with a tendency for decrease with fish (p<0.09) and wheat (p<0.09).

## Conclusion

Female/male differences are observed in EoE: lower BMI, higher risk for malnutrition and more active biological parameters. The elimination diet leads to maintenance of nutritional status, decreased immune activity, identical female/male differences.

## Disclosure of interest

D Colson: Grant/research support from Nutritia Nutrition Clinique, N Kalach: None declared, P Soulaines: None declared, B Michaud: None declared, L Chatenoud: None declared, C Dupont: None declared.

